# The parental intestinal microbiome modulates systemic and local glucocorticoid levels in neonatal mice

**DOI:** 10.1371/journal.pone.0357469

**Published:** 2026-09-23

**Authors:** Jordan E. Hamden, Garrett Ainsworth-Cruickshank, Katherine M. Gray, Jiayu Ye, George V. Kachkovski, Brandon J. Forys, Claire Sie, Jessica A. F. D. Silva, Melody Salehzadeh, Sanjoy Ghosh, Kenneth K. Harder, Kiran K. Soma

**Affiliations:** 1 Department of Biochemistry and Molecular Biology, University of British Columbia, Vancouver, British Columbia, Canada; 2 Djavad Mowafaghian Centre for Brain Health, University of British Columbia, Vancouver, British Columbia, Canada; 3 Department of Biological Sciences, University of Calgary, Calgary, Alberta, Canada; 4 Department of Obstetrics and Gynecology, University of British Columbia, Vancouver, British Columbia, Canada; 5 Department of Biology, University of British Columbia Okanagan, Kelowna, British Columbia, Canada; 6 Department of Emergency Medicine, University of Toronto, Toronto, Ontario, Canada; 7 Department of Psychology, University of British Columbia, Vancouver, British Columbia, Canada; 8 Department of Microbiology and Immunology, University of British Columbia, Vancouver, British Columbia, Canada; 9 Department of Zoology, University of British Columbia, Vancouver, British Columbia, Canada; 10 Graduate Program in Neuroscience, University of British Columbia, Vancouver, British Columbia, Canada; Mae Fah Luang University School of Anti Aging and Regenerative Medicine, THAILAND

## Abstract

Glucocorticoids (GCs) are steroid hormones predominately produced by the adrenal glands and secreted into the blood, but are also produced locally within some target tissues. During early development in mice, postnatal day (PND) 2–12, local GC levels in lymphoid organs are higher than blood GC levels. Interestingly, studies in germ-free (GF) and antibiotic-treated mice demonstrate that the intestinal microbiome modulates circulating and local GC levels, possibly through production of short-chain fatty acids (SCFAs). Importantly, GF mice lack all microbes, and antibiotic treatment is a very powerful perturbation that eliminates most intestinal microbes; the effects of more subtle microbiome manipulations on neonatal blood and local GC levels have yet to be determined. To begin to fill this gap, we established two groups of C57BL/6J mice with distinct microbiomes M1 and M2. We then bred adult mice from M1 and M2 and collected blood, bone marrow, thymus, and spleen from neonatal animals at PND5. We measured progesterone, 11-deoxycorticosterone, corticosterone, and 11-dehydrocorticosterone in the blood and lymphoid organs via liquid chromatography tandem mass spectrometry. We also measured nine SCFAs in the blood to determine a possible connection between microbiome and GCs. The adult microbiomes differed in community composition, but not overall community diversity. Mice with M1 had increased corticosterone and 11-dehydrocorticosteorne levels in blood and lymphoid organs. Mice with M1 also had increased levels of six SCFAs in blood, indicating a possible connection between SCFA signaling and GC levels. These data further our understanding of intestinal microbiome modulation of GC physiology by demonstrating that even subtle differences in microbiome composition can influence systemic and local GC levels.

## Introduction

Glucocorticoids (GCs) are produced by the adrenal cortices and secreted into circulation. Adrenal GC production is tightly regulated by the hypothalamic-pituitary-adrenal axis. Circulating GC levels fluctuate on a circadian cycle and increase in response to stressors. Mice experience a period of adrenal quiescence from postnatal day (PND) 2–12, termed the stress hyporesponsive period (SHRP) [1–4]. During the SHRP, total circulating GC levels are 10–100 fold lower than in adult mice and show very small increases to most stressors, relative to adult animals (humans have an analogous period from ~6 months to 5 years) [5–8].

Some target organs, including lymphoid organs (e.g., bone marrow, thymus, and spleen), can locally produce GCs. Evidence for lymphoid production of GCs includes expression of steroidogenic enzymes, *in vitro* production of corticosterone, high local levels of GCs, and studies of knockout mice [5–7,9–13]. Specifically, within the SHRP, corticosterone levels are elevated in bone marrow, thymus, and spleen, relative to blood [6,12]. In lymphoid organs, GCs modulate B- and T-cell development. For example, during T-cell development, GCs alter thymocyte selection by promoting survival of more reactive T-cells [14]. Less is known about GCs in B-cell development, but B-cells express glucocorticoid receptor (GR), are sensitive to GC-induced apoptosis, and GCs inhibit B-cell receptor signaling *in vitro*, together suggesting a role for GCs in B-cell development [15–18].

Adrenal GC production is regulated by the intestinal microbiome and microbial metabolites such as short-chain fatty acids (SCFAs) [19–21]. Relative to control mice with an intact microbiome, germ-free (GF) mice have higher baseline serum adrenocorticotropic hormone and corticosterone levels, higher serum corticosterone levels in response to stress, and show reduced anxiety- and depression-like behaviors [22–24]. Interestingly, treatment of GF mice with *Bifidobacterium infantis,* a common bacterium in the murine intestine, reduces serum corticosterone to levels in controls [23]. Further, mice treated with antibiotics show increased systemic GC levels compared to controls, and co-housing antibiotic-treated mice with control mice reduces corticosterone levels [20]. Treating conventional mice with *Lactobacillus rhamnonsus* or a cocktail of SCFAs reduces stress-induced corticosterone levels and anxiety- and depression-like behaviours [21,25]. Finally, GF mice treated with an anti-CD3 antibody show decreased local production of corticosterone in the small intestine compared to their SPF counterparts, demonstrating that the intestinal microbiome can modulate local steroid production [26]. Together, these studies demonstrate that very powerful manipulations of the microbiome alter systemic and local corticosterone levels in mice. However, to our knowledge, no study has examined the effects of more subtle intestinal microbiome manipulations, for example, comparing two intact microbiomes that differ in community composition on systemic or local GC levels.

In this study, we compared mice housed side-by-side on the same rack in our animal facility but with two different intestinal microbiomes (M1 and M2), and examined SCFA and steroid levels at PND5. We established two groups of C57BL/6J mice with two distinct microbiomes. We bred the adult mice and collected blood and lymphoid organs from the offspring at PND5 (in the SHRP), to determine the effects of the parental intestinal microbiome composition on neonatal SCFA levels in blood and GC levels in the blood, bone marrow, thymus, and spleen. SCFA levels were measured by gas chromatography mass spectrometry and steroid levels were measured by liquid chromatography mass spectrometry (LC-MS/MS).

## Methods

### Subjects

Subjects were male and female C57BL/6J mice, and PND0 was defined as the first day that pups were present in the cage. 6-week old male and female mice were used for microbiome manipulation and then breeding, neonatal subjects were PND5. PND5 was selected because it is within the SHRP, when lymphoid organ GCs are higher and more sensitive to stressors than blood GC levels. Sex of PND5 mice was determined via genotyping at the University of British Columbia Genotyping Facility.

Mice were housed in a specific pathogen-free colony in the Modified Barrier Facility at the University of British Columbia. Colony rooms were maintained between 20–22°C with 40–70% relative humidity. Mice were housed under a 14:10 light:dark cycle (lights on 0600–2000h) in ventilated Ehret polysulfone Type IIL cages with a CO_2_ membrane, beta-chip bedding, and free access to water (purified by reverse osmosis and sterilized by chlorination) and food (Harland Teklad Global diet 2919 for breeders and diet 2918 after weaning). A red translucent hut and nestlet were placed in each cage for enrichment, and additional crinkle paper was supplied to breeder cages. All procedures were in compliance with the Canadian Council on Animal Care, and protocols were approved by the University of British Columbia Animal Care Committee (A14-0364; A14-0353).

### Microbiome manipulation

Adult (6-weeks) C57BL/6J mice were purchased from the Jackson Laboratory. Upon arrival, mice were housed in soiled cages from mice already housed in this facility with two distinct previously established microbiomes. Mice were exposed to either Microbiome 1 (M1) or Microbiome 2 (M2) for two weeks prior to pairing for breeding. After pairing, mice were always housed in clean cages.

### Tissue collection

PND5 mice were rapidly and deeply anesthetized with 5% isoflurane (2 L/min) and euthanized by rapid decapitation. Euthanasia was completed within 3 min of initial cage disturbance to avoid stress-induced changes in steroids. Trunk whole blood (hereafter “blood”) was collected in 1.5 mL polypropylene microcentrifuge tubes (VWR, Edmonton, AB) and snap frozen on dry ice. Blood was used for steroid measurements to facilitate comparisons between circulating and local steroid levels because serum and plasma overestimate circulating steroid levels [6]. Lymphoid organs were dissected and snap frozen on powdered dry ice. Blood and lymphoid organs were stored at −70°C.

### DNA extraction and 16S sequencing

Feces were collected from adult mice (>PND80) and snap frozen on dry ice. DNA was extracted using the DNeasy Powersoil Kit (QIAGEN, Cat. 12888−100). Briefly, fecal pellets (adults) or entire large intestine, cecum, and contents (neonates) were homogenized in solution C1, 500 µL of supernatant was collected and mixed with solution C2, 600 µL supernatant was collected and mixed with solution C3, 750 µL was collected and mixed with solution C4, 675 µL was collected and added the spin column, flow through was discarded, columns were washed with solution C5, and DNA was eluted using solution C6. 16S amplicons were prepared from DNA by targeting the V4 (adult) and V4-B5^b^ (neonates) regions (Table 1). Amplicons were sequenced on an Illumina Miseq (2x300bp sequencing chemistry) at the Dalhousie IMR (imr.bio) as originally described [27] and updated [28]. Importantly, neonatal samples could not be analyzed due to the relatively low number of bacterial ASVs sequenced from the neonatal intestine, so only adult extracts were amplified and analyzed.

**Table 1:** Primer sequencing for 16S amplification.

	Forward	Reverse
Adult (V4)	515F- GTGYCAGCMGCCGCGGTAA	806R- GGACTACHVGGGTWTCTAAT
Neonate (V4-V5)	515FB- GTGYCAGCMGCCGCGGTAA	926R- CCGYCAATTYMTTTRAGTTT

### SCFA extraction and measurement

30 µl blood samples were mixed with 700 μl of isopropyl alcohol containing 2-ethylbutyric acid at a concentration of 0.01% v/v, which was used as an internal standard. The samples were vortexed at maximum speed for 1 min, then left at room temperature for 15 min. Following incubation, samples were centrifuged at 15,100 × g for 10 min at 4°C. The supernatant was carefully transferred to a gas chromatography vial for subsequent analysis. 1 μl aliquot of the cleared supernatant was injected into a Trace 1300 Gas Chromatograph (Thermo Fisher Scientific) equipped with a flame-ionization detector (FID) and an AI/AS 1310 auto sampler (Thermo Fisher Scientific), operating in splitless mode. Chromatographic separation was achieved using a fused silica FAMEWAX column (30 m × 0.32 mm i.d., 0.25 μm film thickness; Restek, Bellefonte, PA, USA), with helium (Praxair) as the carrier gas at a flow rate of 1.8 mL/min. The oven temperature program was as follows: initial temperature of 80°C, held for 5 min, increased to 90°C at 5°C/min, then increased from 90°C to 105°C at 0.9°C/min, followed by an increase to 240°C at 20°C/min, and held for 5 min. The FID and injection port temperatures were set at 240°C and 230°C, respectively. The flow rates of hydrogen, air, and nitrogen (used as makeup gas) were 30 mL/min, 300 mL/min, and 20 mL/min, respectively. Data were acquired and analyzed using Chromeleon software, version 7.2 (Thermo Fisher Scientific).

A serial dilution of the Volatile Free Acid Mix (CRM46975, Sigma) was prepared to generate standards with the following concentrations: 1.11, 0.37, 0.123, and 0.041 mg/mL. The calibration curve was generated by running each standard under the same conditions described for the samples. Peak areas were extracted from the chromatographic data, and a standard curve was generated by plotting the concentration of the standards against the corresponding peak areas.

### Steroid extraction and measurement

Blood (5 µl; 5.25 mg) was placed in 2 mL polypropylene bead ruptor tubes with 5 zirconium oxide beads. Steroids were extracted from blood and brain tissue via liquid-liquid extraction as previously described. Calibration curves, blanks, and quality controls were processed alongside samples. The calibration curve range was 0.05–1000 pg for all steroids and prepared in 50:50 HPLC-grade methanol:MilliQ water.

Steroid analysis by LC-MS/MS was conducted as previously described. Total progesterone, 11-deoxycorticosterone (DOC), corticosterone, 11-dehydrocorticosterone (DHC), 11-deoxycortisol, cortisol, and cortisone were measured using multiple reaction monitoring with 2 unique mass transitions with unique retention times for each analyte. Steroid concentrations were acquired with a Sciex 6500 Qtrap triple quadrupole tandem mass spectrometer (Sciex LLC, Framingham, MA) in positive electrospray ionization mode for all steroids. The lower limit of quantification was 0.05 pg per sample. Pooled mouse serum was used as an inter-assay control to allow for comparison across all sample runs (n = 3/run, 3 runs total). Deuterated internal standards (progesterone-d9, corticosterone-d8, and cortisol-d4, C/D/N Isotopes Inc., Pointe-Claire, Canada) were included in all standards, blanks, quality controls and samples to correct for losses and matrix effects.

### Data analysis

For PND5 animals, there was no effect of Sex and no interaction between Sex and any other variable for both SCFA and steroid levels (p ≥ 0.12 in all cases) so data from males and females were pooled as before [5,6,9,12,29,30]. One animal was excluded from each group for being greater than 2 standard deviations from the mean. Data are publicly available at: https://doi.org/10.5683/SP3/JNRLAR.

#### Microbiome processing/taxonomy.

Demultiplexed fastq files were processed in Rstudio (v.4.4.2) using the dada2 package (v1.24.0). Dada2 processing consisted of files being primer trimmed (cutadapt v4.1)), quality filtered, dereplicated, and chimera removal. In order to remove amplicon sequence variants (ASVs) — unique, single-nucleotide variations in DNA sequences used to determine bacterial taxonomy — with low reads counts and singletons, ASVs with a relative abundance less than 0.1% were removed. Taxonomy was assigned according to the SILVA v138 database using the dada2 pipeline. Post dada2 processing was done in Rstudio using the phyloseq package (v1.40.0). ASVs identified as chloroplast, mitochondria and eukaryotic as well as sequences unassigned at the domain level were filtered out. Additionally, ASV with count of two less per sample were filtered out to minimize the potential effect of barcode switching and ASVs of less than 250 reads across all samples were filtered out. The filtered phyloseq object was rarefied to the minimum sample ASV count (58011) for community-level analyses. Non-community level analyses were conducted using the non-rarefied phyloseq object.

#### Predicted metabolic pathways.

Biochemical pathways identified from the non-rarefied dataset were annotated using the PICRUSt2 methods (version 2.3.0 beta) and using an available GitHub pipeline [31,32]. Filtering the predicted biochemical pathway phyloseq object was conducted by removing classified pathways with counts less than two per sample and by removing low sequence samples ≤ 250 bp. For community-level analyses, the predicted biochemical pathway dataset was rarefied to the minimum predicted pathway read count per sample (3975518).

#### Microbiome analysis.

Statistical analysis and visualizations were conducted through R. Studio (v.4.2.0) using the base plot package and ggplot2 (v.3.36) (n = 5/group). Community diversity (α-diversity) as measured by Shannon Index (Richness and Evenness) was compared between M1 and M2 using a one-way t-test. To compare differences in bacterial community (β-diversity) as measured by Bray-Curtis dissimilarity index (presence and absence-based), permutational multivariate ANOVA (PERMANOVA) was performed with 999 sample permutations. Non-metric multidimensional scaling (NMDS) plots of Bray-Curtis dissimilarity index were used to visualize community composition. Diversity indexes calculations and PERMANOVA were conducted via the vegan package (v.2.6−2). Relative abundance plots of the top 15 genera were made to visualize differences in specific taxa between groups and DESeq2 analysis (v.1.36.0) was performed to statistically detect differences in specific taxa between groups at the genus level. Analyzing predicted biochemical pathway composition and differences in specific predicted biochemical pathways followed the same methodology as when analyzing taxa.

#### Short-chain fatty acid data analysis.

Data were analyzed for an effect of Microbiome for each SCFA by Mann-Whitney U test (n = 9/group).

#### Steroid data analysis.

A steroid was considered below the lower limit of quantification (LLOQ) if the quantifier and qualifier transitions were not present. For groups with at least 20% of measurements above the LLOQ, the measurements below the LLOQ were replaced with LLOQ/√2. For bone marrow, progesterone was only detectable in 1 animal per group and 11-deoxycorticosterone (DOC) was non-detectable in all animals (Table 2). Thus, bone marrow was excluded from the progesterone and DOC analysis. Data were analyzed for effects of Tissue and Microbiome and for a Tissue x Microbiome interaction for each steroid separately by mixed-effects model (α = 0.05 for all statistical tests). To ensure homogeneity of variance, data were log transformed prior to analysis when necessary. All graphs are presented using non-transformed data as mean ± standard error of the mean (n = 8–9/group). All data were analyzed using GraphPad Prism 10 (version 10.4.2).

**Table 2:** Number of values above the lower limit of quantification for each steroid from Microbiome 1 and Microbiome 2.

	Progesterone		DOC		Corticosterone		DHC	
	M1	M2	M1	M2	M1	M2	M1	M2
Blood	6/9	7/9	6/9	2/9	9/9	9/9	9/9	9/9
Bone Marrow	1/9	1/9	0/9	0/9	9/9	8/9	6/9	0/9
Thymus	9/9	9/9	7/9	3/9	9/9	9/9	9/9	9/9
Spleen	9/9	9/9	9/9	6/9	9/9	9/9	9/9	9/9

Abbreviations: 11-Deoxycorticosterone (DOC); 11-Dehydrocorticosterone (DHC).

## Results

### Adult microbiomes

**Fig 1:**
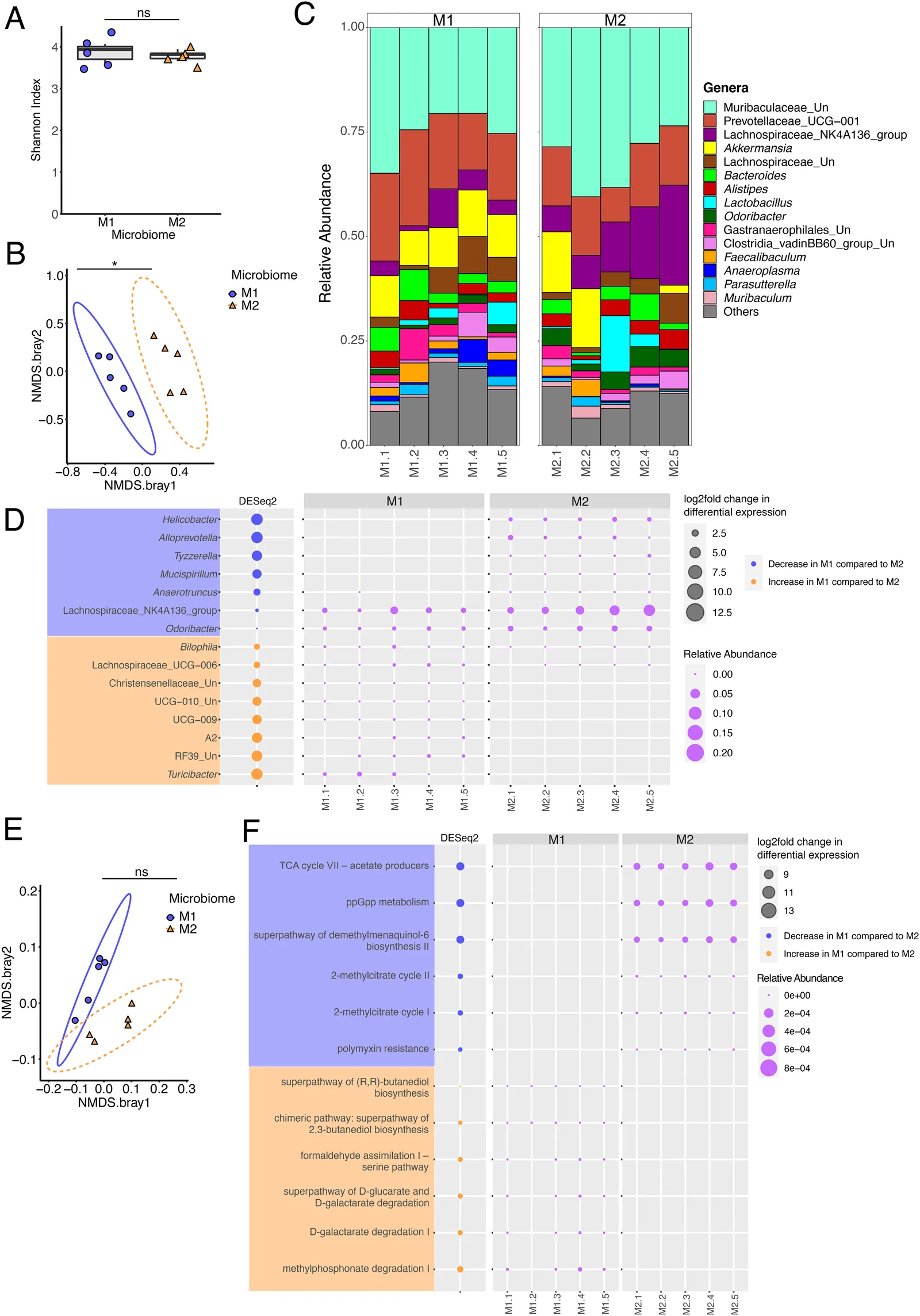
16S sequencing from parental mice showing two distinct microbiomes, Microbiome 1 (M1) and Microbiome 2 (M2). (A-D) Taxonomic analysis. (E and F) Predicted biochemical pathway analysis. (A) α-diversity. Boxplot showing median Shannon (evenness and richness) index to describe the most abundant taxa across both groups, each point represents an individual mouse. (B and E) Taxa (B) and Biochemical pathways (E). β-diversity. Non-metric multidimensional scaling (NMDS) plot in two dimensions of Bray-Curtis dissimilarity (presence and absence-based) index. Each point represents an individual mouse and ellipsoids represent the 95% confidence interval for each group. (C) Relative Abundance barplot of the top 15 genera across samples. (D and F) Taxa (D) and Biochemical pathways (F). Left: DESeq2 results of significant genera that differ between facilities. Color of dot indicates decrease or increase in Microbiome 1 mice compared to Microbiome 2 mice. Size of dot is proportional to the log2fold change in differential abundance. Right: Bubble plot indicating the presence and abundance of each significant genera from DESeq2 across samples. p ≤ 0.05; *, ns; non-significant.

To determine if the fecal microbiome differed between M1 and M2, community level and non-community level analyses were performed (n = 5/group). The overall bacterial diversity (α-diversity) in adult mice from M1 and M2 did not significantly differ (Shannon: t(5.84) = 0.46, p = 0.65, n1 = 5, n2 = 5; Fig 1A). However, a significant difference in community compositions (β-diversity) was detected (Bray-Curtis: Pseudo-F = 4.6, df = 1, R2 = 0.36, p = 0.01; Fig 1B). The differences in community composition were further demonstrated by the relative abundance of the top 15 genera across all microbiome samples in both groups (Fig 1C). We performed DEseq2 to identify what taxa were driving the differences in community composition between groups (Fig 1D). Fifteen genera were differentially abundant between the two groups, with 7 genera higher in M2 (Helicobacter, Alloprevotella, Tyzerella, Mucispirillum, Anaerotruncus, Lachnospiraceae_NK4A136_group, and Odoribacter) and 8 genera higher in M1 (Bilophila, Lachnospiraceae_UCG-006, Christensenellaceae_UN, UCG-010_un, UCG-009, A2, RF39_un, and Turicibacter)*.* Notably, most differences between groups were due to a complete absence of the genera in one group. Only Lachnospiraceae_NK4A136_group and Odoribacter overlapped between the 15 most abundant taxa across all microbiome samples across both groups and those identified as significantly different (Fig 1C and 1D). Predicted biochemical pathway overall composition did not significantly differ between groups (Bray-Curtis: Pseudo-F = 1.03228, df = 1, R2 = 0.11434, p = 0.355; Fig 1E). However, 12 specific biochemical pathways were significantly different between groups (PWY-7254 (TCA cycle VII – acetate producers), PPGPPMET-PWY (ppGpp metabolism), PWY-7373 (superpathway of demethylmenaquinol-6 biosynthesis II), PWY-5747 (2-methylcitrate cycle II), PWY0–42 (2-methylcitrate cycle I), PWY0–1338 (polymyxin resistance), P125-PWY (superpathway of (R,R)-butanediol biosynthesis), PWY-6396 (chimeric pathway: superpathway of 2,3-butanediol biosynthesis), PWY-1622 (formaldehyde assimilation I – serine pathway), GALACTARDEG-PWY (D-galactarate degradation I), GLUCARGALACTSUPER-PWY (superpathway of D-glucarate and D-galactarate degradation), and PWY0–1533 (methylphosphonate degradation I) (Fig 1F).

### Neonatal short-chain fatty acids

Nine short chain fatty acids were quantifiable in the blood of PND5 mice (n = 9/group) (Table 3). There was a significant effect of Microbiome for acetic acid (p = 0.0149), isobutyric acid (p < 0.0001), butyric acid (p < 0.0001), hexanoic acid (p = 0.0018), n-heptanoic acid (p = 0.0015), and isovaleric acid (p = 0.0149). There was no significant effect of Microbiome for formic acid (p = 0.0984), propionic acid (p = 0.4755), or isocaproic acid (p = 0.7448).

**Table 3:** Short chain fatty acid levels in blood of PND5 mice. Data are expressed as mean ±SEM and were analyzed by Mann-Whitney test.

	Microbiome 1(nM)	Microbiome 2 (nM)	Mann-Whitney U p-value
Formic Acid	10.97 ± 1.80	9.08 ± 2.30	0.098
Acetic Acid	4.35 ± 0.29	3.95 ± 0.28	0.015
Propionic Acid	3.37 ± 0.17	3.30 ± 0.12	0.476
Isobutyric Acid	8.41 ± 0.19	8.07 ± 0.07	<0.0001
Butyric Acid	9.92 ± 0.01	9.90 ± 0.004	<0.0001
Isovaleric Acid	10.68 ± 0.12	10.56 ± 0.07	0.015
Isocaproic Acid	7.49 ± 0.06	7.47 ± 0.01	0.74
Hexanoic Acid	5.40 ± 0.14	5.27 ± 0.04	0.002
n-Heptanoic Acid	7.68 ± 0.17	7.55 ± 0.02	0.002

Note; n = 9/group.

### Neonatal steroids

**Fig 2:**
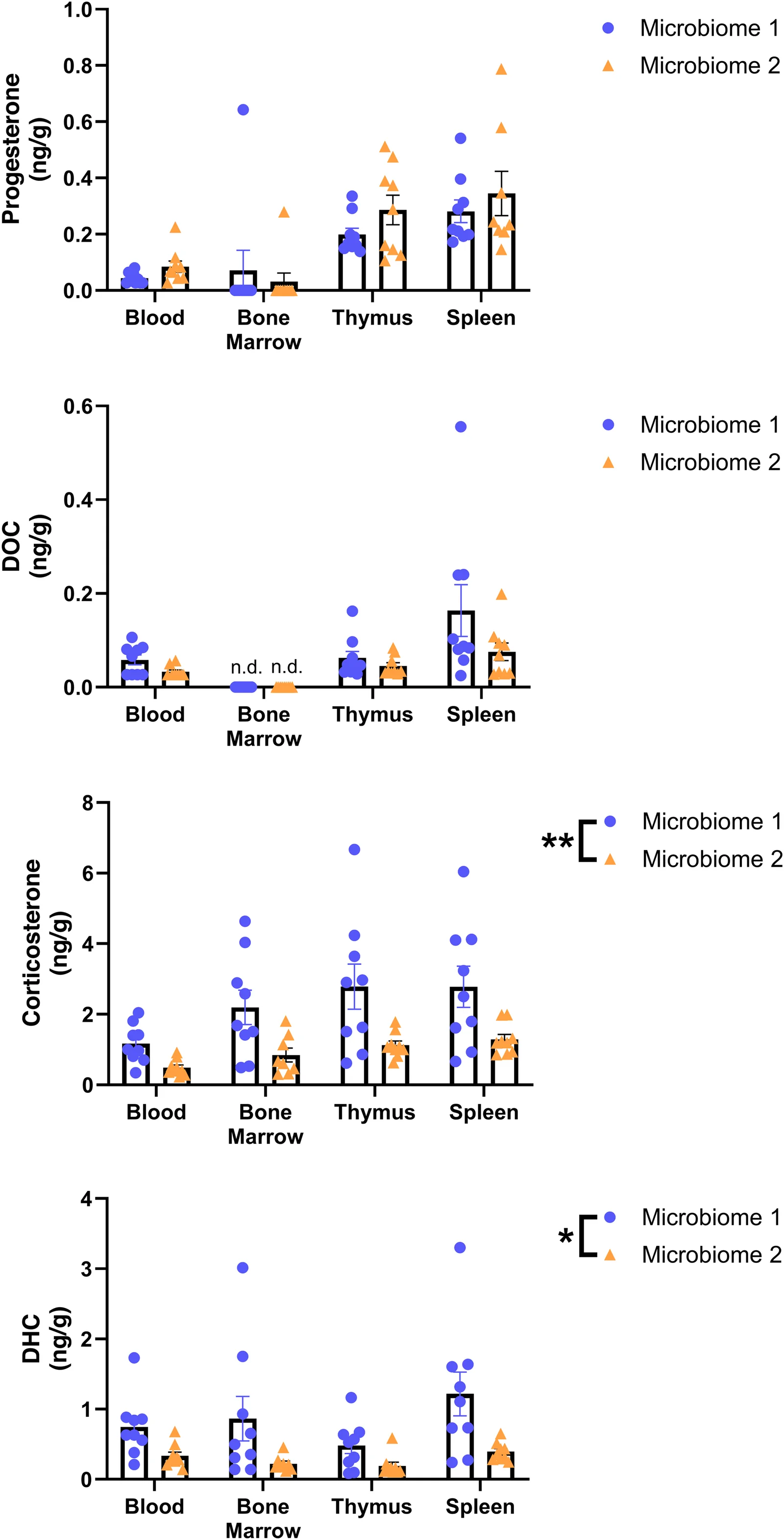
Steroid concentrations in blood and lymphoid organs of neonatal mice. (A) progesterone, (B) 11-deoxycorticosterone (DOC), (C) corticosterone, (D) 11-dehydrocorticosterone (DHC) in whole blood, bone marrow, thymus, spleen of postnatal day 5 mice with Microbiome 1 or Microbiome 2. Data were analyzed by Mixed-effects model for all steroids. Data are shown as mean ± SEM. n = 8-9 for all groups.

Progesterone was above the LLOQ in only 2 bone marrow samples and DOC was below the LLOQ in all bone marrow samples, and thus bone marrow was excluded from analyses for progesterone and DOC (Table 2). For progesterone and DOC, there were significant main effects of Tissue only (p < 0.0001 for both steroids) (Fig 2). For corticosterone, there were significant main effects of Tissue (p < 0.0001) and Microbiome (p = 0.0095), but no Tissue x Microbiome interaction (p = 0.3267). For DHC, there were significant main effects of Tissue (p < 0.0001) and Microbiome (p = 0.0113), but no Tissue x Microbiome interaction (p = 0.7579). 11-deoxycortisol, cortisol, and cortisone were non-detectable in all samples.

## Discussion

In this study, we established two groups of C57BL/6J mice (in the same room) with two distinct microbiomes, profiled the adult microbiome, and measured neonatal blood SCFA levels and blood and lymphoid GC levels. It has been demonstrated that GF and antibiotic-treated mice have higher circulating and intestine GC levels than mice with an intact microbiome, but this is the first study to demonstrate that mice with microbiomes that differ in beta diversity have different blood and lymphoid GC levels. This is also the first study to show that the parental microbiome modulates GC levels in early postnatal development, a time of critical importance for lymphocyte development, which is strongly modulated by GCs [33].

Unfortunately, we could not assess the neonatal microbiome in this study because of the relatively low bacterial load in neonatal mice compared to adults. Due to the lack of neonatal microbiome data, all relationships between the adult microbiome and neonatal SCFA and GC levels are associative. Several studies have attempted to profile the microbiome in neonatal mice from birth to weaning. Pantoja-Feliciano et al. (2013) demonstrated that 1 day after birth, neonatal colonic microbiome more closely resembles the dam vaginal microbiome than the dam fecal microbiome [34]. The PND1 microbiome is primarily composed of *Streptococcaceae* and various taxa from the Proteobacteria phylum. By PND3 and PND9, neonatal colonic microbiome diversity decreases sharply, becoming dominated by *Lactobacillus, Streptococcus*, and *Pasteurella*. By PND21, colonic microbiome diversity increases to more closely mirror that of the dams, which is thought to occur as the pups become coprophagic. A study by van Best et al. (2020) similarly found a dominance of *Lactobacillus* at PND7, with microbiome composition becoming more “adult-like” by PND21 [35].

Until recently, the dynamics and source of the microbes in the neonatal microbiome were not well understood. A recent study by Kennedy et al. (2025) sequenced pup fecal samples from PND4-PND9, then at PND11, 13, 15, 21, and into adulthood [36]. Similar to previous studies, the PND4–14 microbiome is dominated by *Ligilactobacillus* (a newly described genus that was previously included in the *Lactobacillus* genus) and *Streptococcus*, which are rarely found in maternal fecal samples and other body sites*.* When comparing the pup and dam microbiome composition, similarity was lowest before PND10, and by PND15–20, the pup microbiome was more similar to its own dam’s microbiome than other dams [36]. Overall, the neonatal mouse gut microbiome is compositionally and functionally distinct from the adult mouse gut microbiome. The dominance of *Ligilactobacillus* and *Lactobacillus* in the neonatal mouse microbiome is widely thought to be due to their ability to ferment breast milk sugars such as lactose [37]. Although there is some evidence that specific strains of *Lactobacillus* spp. can produce SCFAs, they are not typically considered canonical SCFA producers [38].

The two adult microbiomes that we established in this study did not differ in alpha diversity, but did differ in beta diversity, a measure of community composition. In adult microbiomes, only two significantly different taxa between M1 and M2 were among the 15 most abundant taxa in both microbiomes, suggesting that differences in genera abundance are driven largely by lower abundance taxa and by presence in one microbiome only, rather than shifts in abundance between microbiomes. For example, taxa from *Helicobacter, Alloprevotella, Tyerella, and Mucispirillum* were present in all M2 samples and absent in all M1 samples. Interestingly, all genera, excluding *Biophilia*, that were present in M1 but absent in M2 (Christensenellaceae_Un, UCG-010_Un, UCG-009, A2, RF39_Un, and *Turicibacter*) belong to the phylum *Firmicutes*, which are among the main SCFA producers in the murine intestine. Predicted metabolic pathway analysis revealed no overall differences in pathway composition; however, specific pathways varied between M1 and M2. While these results are predictive, they suggest a functional shift in the microbiome, corresponding to observed taxonomic changes. Direct assessment of the microbiome’s metabolomic profile, using shotgun metabolomics, alongside blood and brain metabolite quantification may further elucidate the relationship between the microbiome, SCFAs, and GCs.

Compared to M2 neonates, M1 neonates had significantly higher blood levels of six SCFAs, including acetate and butyrate. Interestingly, acetate, butyrate, and propionate decreased corticosterone levels in stressed adult mice when given in drinking water [21]. As treatment with SCFAs decreased corticosterone levels in adults, it might be expected that neonates with higher blood SCFA levels would have lower blood corticosterone levels. However, M1 neonates had both higher SCFA and GC levels. The discrepancy between previous reports and the present data may be due to a difference in age. Previous studies have conducted experiments in adult animals, and to our knowledge, these are the first data examining the relationship between SCFA and GC levels in neonatal animals. Alternatively, experiments that treat animals with exogenous SCFAs artificially elevate circulating SCFA levels, and experiments in GF and antibiotic-treated animals dramatically reduce circulating SCFA levels. It is possible that the corticosterone-suppressive effects of SCFAs are less pronounced at physiological SCFA levels. However, more mechanistic study is needed to determine a causal relationship between SCFAs and GC levels in neonatal animals. Compared to SCFA levels in adult mice, these neonatal blood levels are considerably lower and less variable across SCFAs [39]. This large difference in neonatal SCFA levels compared to adult levels is likely due to reduced SCFA-producing bacteria in the neonatal intestine. Interestingly, microbiota-produced maternal SCFAs can reach embryos during pregnancy and are delivered through breast milk during neonatal development, SCFAs can be synthesized by neonates through breakdown of carbohydrates present in breast milk [40–42]. Thus, the higher blood SCFA levels in M1 neonates may be due to maternal microbiome.

Blood GC levels in M1 and M2 neonates were low, consistent with the SHRP, but higher in M1 than M2 [5–10,12]. Lymphoid GC levels were also similar to previously reported levels in neonatal mice [6,7,10,12] and also higher in M1 than M2. Additionally, GC levels were higher in the thymus than the bone marrow or spleen in both groups. These are the first data demonstrating that the intestinal microbiome can modulate local GC levels within lymphoid organs. GCs affect the immune system and lymphocyte development [43–47]. However, these studies used repeated stressors that produce large increases in GC levels (e.g., lipopolysaccharide) or used in vitro models that cannot be easily compared to in vivo GC levels. It is unclear how more subtle changes in GC levels during early postnatal development alter lymphoid system development in neonatal animals and function in adult animals. However, increased GC levels during lymphoid system and cell development generally lead to a more reactive lymphoid cell repertoire [47].

Studies in GF and antibiotic-treated mice indicate that the intestinal microbiome can modulate blood and intestinal GC levels, possibly through microbial SCFAs. The present data also support the idea that the parental intestinal microbiome modulates neonatal GC levels. These are the first data to demonstrate that more subtle changes in the intestinal microbiome (compared to antibiotic treatment) modulate both blood and lymphoid organ GC levels in neonatal animals. Interestingly, animals from M1 and M2 also had different neonatal blood SCFA levels. These differences in circulating SCFA levels provide a possible mechanism for the intestinal microbiome to modulate neonatal GC levels.
