# Correction: The parental intestinal microbiome modulates systemic and local glucocorticoid levels in neonatal mice

**DOI:** 10.1371/journal.pone.0361477

**Published:** 2026-10-23

**Authors:** Jordan E. Hamden, Garrett Ainsworth-Cruickshank, Katherine M. Gray, Jiayu Ye, George V. Kachkovski, Brandon J. Forys, Claire Sie, Jessica A. F. D. Silva, Melody Salehzadeh, Sanjoy Ghosh, Kenneth W. Harder, Kiran K. Soma

The 11th author’s name is spelled incorrectly. The correct name is: Kenneth W. Harder.
